# PPARβ/δ accelerates bone regeneration in diabetic mellitus by enhancing AMPK/mTOR pathway-mediated autophagy

**DOI:** 10.1186/s13287-021-02628-8

**Published:** 2021-11-04

**Authors:** Miao Chen, Dian Jing, Rui Ye, Jianru Yi, Zhihe Zhao

**Affiliations:** 1 State Key Laboratory of Oral Diseases and National Clinical Research Center for Oral Diseases, West China Hospital of Stomatology, Sichuan University, Chengdu, China; 2 Department of Orthodontics, West China Hospital of Stomatology, Sichuan University, No. 14, 3rd Section, South Renmin Road, Chengdu, 610041 Sichuan China; 3 Department of Orthodontics, Shanghai Ninth People’s Hospital, Collage of Stomatology, Shanghai Jiao Tong University School of Medicine, Shanghai, China

**Keywords:** PPARβ/δ, Diabetes mellitus, Autophagy, Osteogenic differentiation, Bone regeneration, AMPK/mTOR pathway

## Abstract

**Background:**

Diabetic patients are more vulnerable to skeletal complications. Peroxisome proliferators-activated receptor (PPAR) β/δ has a positive regulatory effect on bone turnover under physiologic glucose concentration; however, the regulatory effect in diabetes mellitus has not been investigated yet. Herein, we explored the effects of PPARβ/δ agonist on the regeneration of diabetic bone defects and the osteogenic differentiation of rat bone marrow mesenchymal stem cells (rBMSCs) under a pathological high-glucose condition.

**Methods:**

We detected the effect of PPARβ/δ agonist on osteogenic differentiation of rBMSCs in vitro and investigated the bone healing process in diabetic rats after PPARβ/δ agonist treatment in vivo. RNA sequencing was performed to detect the differentially expressed genes and enriched pathways. Western blot was performed to detect the autophagy-related protein level. Laser confocal microscope (LSCM) and transmission electron microscope (TEM) were used to observe the formation of autophagosomes.

**Results:**

Our results demonstrated that the activation of PPARβ/δ can improve the osteogenic differentiation of rBMSCs in high-glucose condition and promote the bone regeneration of calvarial defects in diabetic rats, while the inhibition of PPARβ/δ alleviated the osteogenic differentiation of rBMSCs. Mechanistically, the activation of PPARβ/δ up-regulates AMPK phosphorylation, yielding mTOR suppression and resulting in enhanced autophagy activity, which further promotes the osteogenic differentiation of rBMSCs in high-glucose condition. The addition of AMPK inhibitor Compound C or autophagy inhibitor 3-MA inhibited the osteogenesis of rBMSCs in high-glucose condition, suggesting that PPARβ/δ agonist promotes osteogenic differentiation of rBMSCs through AMPK/mTOR-regulated autophagy.

**Conclusion:**

In conclusion, our study demonstrates the potential role of PPARβ/δ as a molecular target for the treatment of impaired bone quality and delayed bone healing in diabetic patients for the first time.

**Supplementary Information:**

The online version contains supplementary material available at 10.1186/s13287-021-02628-8.

## Background

Diabetes mellitus is a metabolic disease characterized by hyperglycemia that can lead to chronic damage to various organs and tissues, further causing profound consequences on patients’ quality of life. Due to the incessantly increasing incidence in the past decades, diabetes mellitus has become a global public problem that endangers human health [1]. According to the World Health Organization’s report on diabetes mellitus, the number of adults living with diabetes worldwide was 422 million in 2014, which was estimated to rise to 550 million by 2030 [2, 3]. The occurrence of diabetes mellitus has increased rapidly in developing countries during the past 10 years along with the urbanization process and changes in lifestyle. Currently, China has become the country with the most diabetic patients worldwide[4].

Diabetic patients are more vulnerable to skeletal complications, which are called as “diabetic bone disease” or “diabetic osteopathy” [5]. The common diabetic bone diseases include osteoporosis, increased fracture risk and poor bone healing properties [6]. Numerous studies demonstrated that the fracture risk in diabetic patients is higher than that in healthy individuals [7–9]. In addition, the healing time of fracture in diabetic patients would be greatly prolonged [10, 11]. The molecular modulation of bone metabolism in diabetic environment has also been extensively explored. The hyperglycemia condition inhibits the maturation and metabolism of mesenchymal stem cells (MSCs) [12, 13]. The pathologically high-glucose environment has been proven to promote osteoblast apoptosis and inhibit osteogenic differentiation. In diabetic patients, the increased osteoblast apoptosis led to decreased bone remodeling activity [14, 15]. The aforementioned pathological mechanisms ultimately result in bone loss and impaired bone quality. Effective therapeutic methods to promote the bone regeneration in hyperglycemia environment are essential to improve the life quality of patients with diabetic bone diseases.

Peroxisome proliferators-activated receptor (PPAR) β/δ is a type of ligand-activated receptor, belonging to nuclear-receptor superfamily. When activated by endogenous ligands or artificial agonists, PPARβ/δ acts as a transcription factor through binding to the peroxisome proliferator response elements (PPREs) located in the promoter region of targets genes [16]. PPARβ/δ controls a multitude of metabolic processes including lipid catabolism, glucose homeostasis, redox balance, inflammation and differentiation [17, 18]. Additionally, PPARβ/δ has therapeutic potential to promote the mammalian regeneration of bone, skin, muscle and liver [16]. The regulatory effect of PPARβ/δ on bone turnover was firstly recognized in 2013 [19]. Conditional knockout of PPARβ/δ in mice via Runx2-cre led to the energy metabolism disorder in osteoblasts, thus impaired the bone mineralization and bone mass [20]. Absence of PPARβ/δ led to glucose intolerance and impaired bone formation [21]. These studies provide a new mechanism to explain the increased fracture risk in diabetes and consider PPARβ/δ as a potential molecular target for the treatment of bone fragility in diabetes. In addition, the activation of PPARβ/δ accelerated osteoblast differentiation and increased the number of peroxisomes, which were ubiquitous organelles in eukaryotic cells to regulate ROS levels [22]. Furthermore, PPARβ/δ has been found to restore the metabolic homeostasis through enhancing insulin sensitivity and regulating glucose metabolism in diabetic mice [23, 24]. Taken together, PPARβ/δ seems to be a potential therapeutic target to treat the impaired bone formation in diabetes. However, this hypothesis has not been investigated yet.

The objective of the present study was to determine the effects of PPARβ/δ on bone regeneration in diabetic condition. In this study, we firstly studied the osteogenic differentiation effect of PPARβ/δ agonist on rat bone marrow mesenchymal stem cells (rBMSCs) in hyperglycemia environment. Secondly, we established a rat calvarial defect model to evaluate the effects of PPARβ/δ agonist on the healing of bone defects in diabetes. Thirdly, we explored the mechanisms of PPARβ/δ-mediated osteogenesis in hyperglycemia condition based on RNA sequencing analysis and performed further validation for the potential mechanisms.

## Materials and methods

### Experimental animals

A total of 32 Sprague–Dawley (SD) rats at an age of 7 weeks were used. These rats were housed in a pathogen-free environment with access to standard rat food and plain water ad libitum under a 12 h light/dark cycle. After a 1-week acclimatization, the SD rats were randomly divided into the following four groups of eight rats each: (1) diabetes mellitus group (DM); (2) diabetes mellitus treated with PPARβ/δ agonist GW501516 group (DM + GW); (3) control group (Control) and (4) control treated with GW501516 (Control + GW).

DM + GW group and Control + GW group were injected with GW501516 (5 mg/kg/d) dissolved in 0.1 ml dimethyl sulfoxide (DMSO) every other day [25]. DM and Control group were injected with 0.1 ml DMSO every other day as control. The rats were sacrificed 4 and 8 weeks after surgery. The samples were harvested for histology analysis and μCT assay.

DM model was induced by intraperitoneal injection of 1% streptozotocin (STZ) solution with a dose of 60 mg/Kg. The STZ solution was prepared by dissolving STZ (Sigma-Aldrich) in 0.1 mM citric acid-citrate sodium buffer (pH 4.5) [26]. The citric acid-citrate sodium buffer without STZ was intraperitoneally injected into normal rats as control. The experimental rats were fasted for 12 h before STZ administration. The blood glucose of rats after a 12-h fasting was monitored on day 7 and 14 after streptozotocin injection. The rats with a fasting blood sugar values higher than 16.65 mmol/L were considered eligible [26].

Calvarial defect model was established in all experimental rats. Each rat was anesthetized with 4% (w/v) isoflurane, followed by an intraperitoneal injection of ketamine (60 mg/kg, Sigma-Aldrich) and xylazine (12 mg/kg, Sigma-Aldrich) [27]. Before the incision was made, bupivacaine (0.25%, 1–2 mg/kg dose) was used to relieve local pain. The surgical area was shaved, and a 1 cm midline skin incision was made between the rats’ ears. Periosteum were separated to fully expose the calvarial bone. The bone defect areas with a diameter of 3 mm on both sides of cranial suture were prepared by dental trephine under normal saline rinsing [28]. The periosteum and skin incisions were sutured in layers with absorbable sutures after surgery.

### Micro-computed tomography (μCT) and histomorphometrical analyses

Calvarial bone were dissected and fixed in 4% paraformaldehyde for 2 days and then stored in 70% ethanol at 4 °C. The μCT analysis was performed (μCT50, SCANCO Medical) with a spatial resolution of 10 μm (55 kV, 114 mA, 500 ms integration time). The region of interest (ROI) was defined as a cylindrical area covering the initial bone defect with a diameter of 3 mm to analyze the regeneration of cortical bone [29]. Bone mineral density (BMD) and bone volume/total volume (BV/TV) were evaluated within the delimited ROI [30].

### Histology staining

Following μCT analysis, the samples were decalcified with 10% EDTA for 4 weeks. Sections of 4.5 μm were prepared using a conventional method [31]. H&E and Masson trichrome staining (Solarbio, Beijing, China) were performed as the manufacturer’s instruction. The stained sections were observed using an inverted microscope (IX81, Olympus).

### Cell culture

The rBMSCs were obtained from Cyagen Biosciences. The femur and tibia of sacrificed 4-week-old rats were separated after soaking in 75% ethanol for 5 min. The two ends of epiphysis were cut off to flush out the bone marrow, and the suspension was centrifuged. The supernatant was discarded, and the cells were transferred to DMEM (Gibco, Grand Island, NY, USA) with 5.6 mM glucose concentration containing 10% fetal bovine serum (Hyclone Laboratories, Logan, UT, USA), 2 mM L-glutamine (Sigma), 100 U/mL penicillin and 100 mg/mL streptomycin (Hyclone) at 37 °C in 5% CO_2_. The medium was changed every 3 days to discard non-adherent cells [32]. The cells were expanded when the confluency reached approximately 80%. The cells from passage 2 to passage 4 were used in this study.

To obtain the bone marrow-derived macrophages (BMDMs), the bone marrow was flashed into a petri dish and centrifuged as previous described. The supernatant was discarded, and the red blood cell lysate was added. The cells were seeded in a 10-cm-diameter petri dish, cultured in DMEM medium with 10% fetal bovine serum (FBS), and supplied with 50 ng/ml m-CSF (R&D). After 4 days, the suspension cells were discarded and BMDMs were obtained for subsequent experiment.

For osteogenic differentiation, rBMSCs were induced with medium containing 10 mM β-glycerophosphate, 10^–8^ M dexamethasone and 50 μg/ml L-2-ascorbic acid (Sigma-Aldrich, St. Louis, MO, USA) [33]. The culture cells were randomized into normal-glucose (NG) group (5.6 mM) and high-glucose (HG) group (30 mM). The HG medium was obtained by adding D-glucose (Sigma-Aldrich) to the NG medium. The NG and HG group were then divided into three subgroups and added with 0 μM, 1 μM, or 5 μM of PPARβ/δ agonist GW501516 (Sigma-Aldrich), respectively, yielding a total of six groups: N0 (5.6 mM glucose), N1 (5.6 mM glucose + 1 μM GW501516), N5 (5.6 mM glucose + 5 μM GW501516), H0 (30 mM glucose), H1 (30 mM glucose + 1 μM GW501516), and H5 (30 mM glucose + 5 μM GW501516).

### Alkaline phosphatase staining

After 7 days of osteogenic induction, alkaline phosphatase (ALP) staining was performed using a BCIP/NBT ALP color development kit (Beyotime, C3206, China) following manufacturer’s instruction. Briefly, after removing the culture medium, the cells were washed three times with PBS and fixed with 4% polyoxymethylene for 15 min. Then, the cells were immersed by BCIP/NBT staining working solution and incubated at room temperature for 30 min in dark. The color reaction was terminated by distilled water. ALP quantification was performed with an alkaline phosphatase assay kit (Beyotime, P0321M, China) according to the manufacturer’s protocol. The optical density (OD) was evaluated by measuring the absorbance at 405 nm spectrophotometrically. The total protein content was measured at OD_562nm_, and the concentration was calculated according to the standard BSA curve using the enhanced BCA protein assay (Beyotime, P0010) [33]. ALP activity was represented by the OD_405nm_ value after normalization to the total cellular protein.

### Alizarin red S staining and mineralization assay

After 21 days of osteogenic induction, calcium deposition was determined by alizarin red S (ARS) staining and semi-quantitative analysis [33]. The cells were fixed with 4% paraformaldehyde for 15 min and then stained with 0.1% ARS (pH 4.2, Sigma-Aldrich) for 30 min. The stained cells were observed under an inverted microscope (Olympus). The stained mineralized nodules were incubated with 100 mM cetylpyridinium chloride in 10 mM sodium phosphate (pH 7.0) for 1 h to quantify the calcification. The calcium deposition was determined by measuring the absorbance at OD_562nm_ spectrophotometrically.

### Osteoclast induction

For osteoclast induction, 50 ng/mL RANKL (R&D) was added to the BMDM culture medium, and the medium was changed every 2 days. After 3 days of culture, mature osteoclasts could be observed and subsequent experiments were performed.

TRAP staining (Sigma-Aldrich) was performed according to the previously described procedure [34]. The cells were fixed with 4% paraformaldehyde for 15 min, added with TRAP staining solution, and incubated at 37 °C for 40 min. TRAP-positive cells containing 3 or more nuclei were considered osteoclasts.

### Quantitative reverse transcription polymerase chain reaction (qRT-PCR)

The total RNA of rBMSCs was extracted using Trizol reagent (Invitrogen, Carlsbad, CA, USA) and then reversely transcribed to obtain stable cDNA using PrimeScript™ RT reagent Kit with gDNA Eraser (TaKaRa Bio, Otsu, Japan). The qRT-PCR was performed using SYBR Premix Ex Taq II (TaKaRa Bio) in Quant Studio™ 3 real-time fluorescent quantitative PCR instrument (ThermoFisher Scientific, China). Glyceraldehyde 3-phosphate dehydrogenase (*Gapdh*) was used as an internal reference to normalize the gene expression [30]. The result was calculated using the 2^−ΔΔCt^ method and expressed as a multiple change relative to GAPDH. The primer sequences are summarized in Additional file 2: Table S1.

### Flow cytometry

After 7 days of osteogenic induction, rBMSCs were harvested in Dulbecco’s phosphate buffered saline and incubated for 15 min at 4 °C with fluorescent dyes conjugated anti-rat CD29, CD44, CD34 and CD45 (BD Biosciences) protected from light. The apoptosis in N0, H0, H1, and H5 group was determined to evaluate the effect of PPARβ/δ on cell apoptosis in high-glucose environment. After 7 days of culture, the cells in each group were digested and centrifuged. The cells were resuspended in 500 μL of the binding buffer and stained with Annexin V to label the apoptotic cells for 10 min. Then, the PI solution was added to label the dead cells according to the PI-Annexin V apoptosis detection kit (BD Bioscience) [35]. After adjusting the cell suspension volume to 1 mL, the samples were assessed on a flow cytometer (ThermoFisher Scientific).

### Western blot

After 7 days of osteogenic induction, the total proteins were extracted by RIPA buffer (Pierce, Rockford, IL) on ice. Equal quantities of protein samples were separated by electrophoresis on 12% SDS-PAGE polyacrylamide gels. Then, the samples were electro-transferred to PVDF membranes (0.22 μm, Millipore) using a wet transfer apparatus (Bio‐Rad) and blocked with 5% BSA in PBS for 1 h at room temperature. The membranes were incubated overnight at 4 °C with primary antibodies of AMPK (#ET1608-40, huabio, 1:1000), p-AMPK (#2535 T, CST, 1:1000), mTOR (#ET1608-5, huabio, 1:1000), p-mTOR (#ABP50363, abbkine, 1:1000), p62 (#R1309-8, huabio, 1:1000), LC3BI/II (#ET1701-65, huabio, 1:1000) and β-ACTIN(#MA1210-1, huabio, 1:2000), respectively. After that, the blots were incubated with horseradish peroxidase (HRP)-conjugated secondary antibodies (#HA1001, huabio, 1:5000) at room temperature for 1 h [30]. The immobilon reagents (Millipore) were used for the visualization and detection of antibody-antigen complexes. The band intensity was measured by ImageJ software.

### RNA-seq and gene set enrichment analysis

The rBMSCs cultured in high-glucose osteogenic differentiation medium for 7 days were collected for RNA sequencing. Trizol reagent (Invitrogen) was used to extract the total RNA. Sequencing library was prepared according to the steps recommended by the Illumina TrueSeq mRNA sample preparation kit. Sequencing was performed on Illumina HiSeq 3000. STAR software was used to map the reads to the rat genome to obtain a gene expression matrix. DESeq2 package was used to perform the differential gene analysis. Genes with adjusted p value lower than 0.01 and |log2FoldChange| higher than 1.5 were considered statistically significant. KEGG enrichment analysis was performed using the online analysis tool metascape (http://metascape.org/). Gene Set Enrichment Analysis (GSEA) was carried out through GSEA software (http://www.broad.mit.edu/GSEA, v.4.1.0). The two gene sets of “osteogenesis” and “positive regulation of autophagy” were enriched and analyzed.

### Adenoviral infection

The adenoviral RFP-GFP-LC3 (Hanbio, Shanghai, China) was used to transfect rBMSCs to indicate autophagy flux. In brief, 20 MOI virus solution was added to each well on the 5th day since osteogenic induction. After a 6-h infection, the medium containing virus solution was replaced by fresh culture medium. After another 48-h incubation, rBMSCs were washed with PBS and fixed with 4% paraformaldehyde for 15 min. DAPI (Invitrogen) was used to mark nuclei. Autophagosomes were visualized by a laser confocal microscope (LSCM, Olympus).

### Transmission electron microscope (TEM)

The number and morphology of autophagosome were observed using a transmission electron microscope (TEM, Hitachi, Japan). Briefly, the cells of each group were digested and centrifuged. Then, the cell mass at the bottom was collected and fixed with 2.5% glutaraldehyde. After fixation, the cell mass was treated with 1% osmium acid for 2 h, and dehydrated by ethanol and acetone. Then the sample was embedded with epoxy resin to prepare ultrathin slice (50–70 nm). The slice was stained with uranyl acetate-lead citrate, and the autophagosome was observed by TEM.

### Statistical analysis

All quantified data were expressed as mean ± standard deviation (SD). Statistical differences were performed via one-way or two-way analysis of variance (ANOVA) followed by the Tukey’s post hoc test for multiple comparisons. *p*  < 0.05 was considered to be statistically significant.

## Results

### PPARβ/δ agonist promotes osteogenic differentiation and decreases the apoptosis in high-glucose environment

The rBMSCs characterization was performed by three-line differentiation and flow cytometry. ARS, Alcian Blue and Oil Red O staining were conducted to evaluate the osteogenic, chondrogenic and adipogenic differentiation potential of rBMSCs (Additional file 1: Figure S1A-C). Flow cytometry showed that the rBMSCs were CD29/CD44 positive and CD34/CD45 negative (Additional file 1: Figure S1D).

**Fig. 1:**
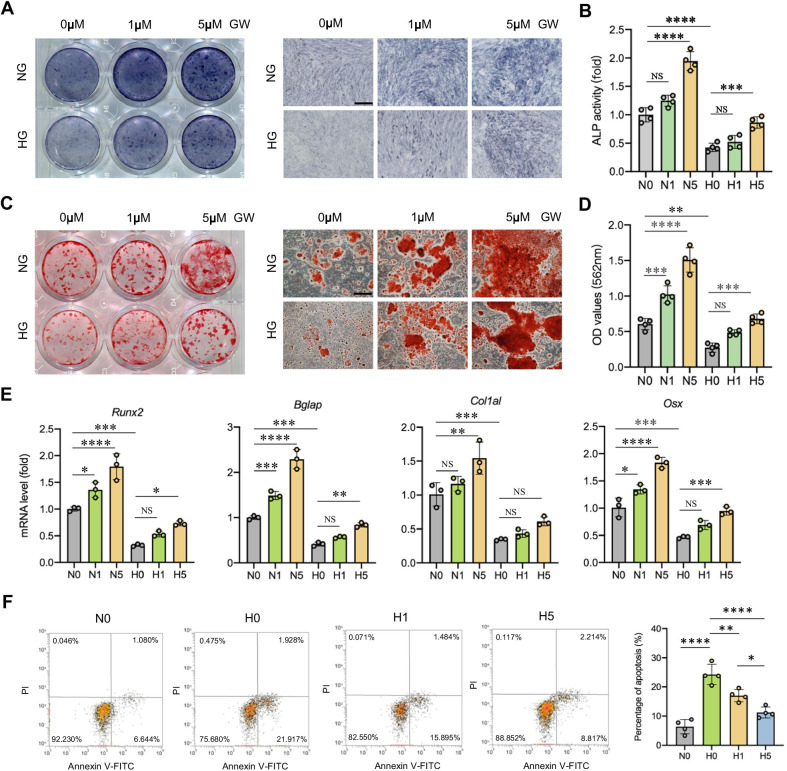
PPARβ/δ agonist promotes osteogenic differentiation and decreases the apoptosis in high-glucose environment. **A**, **B** ALP staining and ALP activity quantitative analyses of rBMSCs after 7 days of osteogenic induction treated with GW501516 of different concentration (scale bar = 500 µm). **C**, **D** ARS staining and quantitative analyses of rBMSCs after 21 days of osteogenic induction treated with GW501516 of different concentration (scale bar = 500 µm). **E** qRT-PCR for the osteogenesis-related genes *Runx2*, *Bglap*, *Col1a1* and *Osx* after 7 days of osteogenic induction. **F** Analysis of cells stained with Annexin V/PI by flow cytometry. Data are expressed as mean ± SD. The *p* values were calculated by two-way ANOVA with Tukey’s post hoc test. (NS, not statistically significant, *p < 0.05, **p < 0.01, ***p < 0.001, ****p < 0.0001)

First, we explored the effects of PPARβ/δ agonist on the osteogenic differentiation of rBMSCs in high-glucose condition. The ALP staining of rBMSC in HG group was lighter than that in NG group. The staining depth increased with addition of GW501516 in a concentration-dependent manner (Fig. 1A, B). Similar results were observed in ARS staining (Fig. 1C, D). The mRNA expressions of osteogenic markers were decreased in high-glucose medium. The addition of GW501516 partially restored the mRNA expressions of osteogenic markers (Fig. 1E). To investigate whether PPARβ/δ agonist-mediated promotion of osteogenic differentiation of rBMSCs was specific to GW501516, we explored the osteogenic effect of another PPARβ/δ agonist, GW0742, in rBMSCs. Similar to GW501516, GW0742 increased ALP staining depth and mRNA expressions of osteogenesis-related genes in normal- and high-glucose conditions (Additional file 1: Figure S2A-D). The addition of PPARβ/δ antagonist GSK0660 inhibited the osteogenic differentiation of rBMSCs in normal- and high-glucose conditions (Additional file 1: Figure S2E-G). The high-glucose condition enhanced the apoptotic cells from 6.36% ± 2.11% to 24.25% ± 3.04%, which was reduced to 17.03% ± 1.79% and 11.22% ± 1.62% by adding 1 μM and 5 μM of GW501516, respectively (Fig. 1F).

In addition, we explored the regulatory effect of PPARβ/δ agonist on osteoclast differentiation in normal- and high-glucose conditions. As previously reported, high-glucose environment inhibited osteoclast differentiation in vitro [36]. Trap staining results showed that there is no significant difference on the number and size of osteoclasts before and after PPAR β/δ agonist treatment in vitro (Additional file 1: Figure S3A, B).

### PPARβ/δ agonist improves the bone regeneration of calvarial defects in diabetic rats

**Fig. 2:**
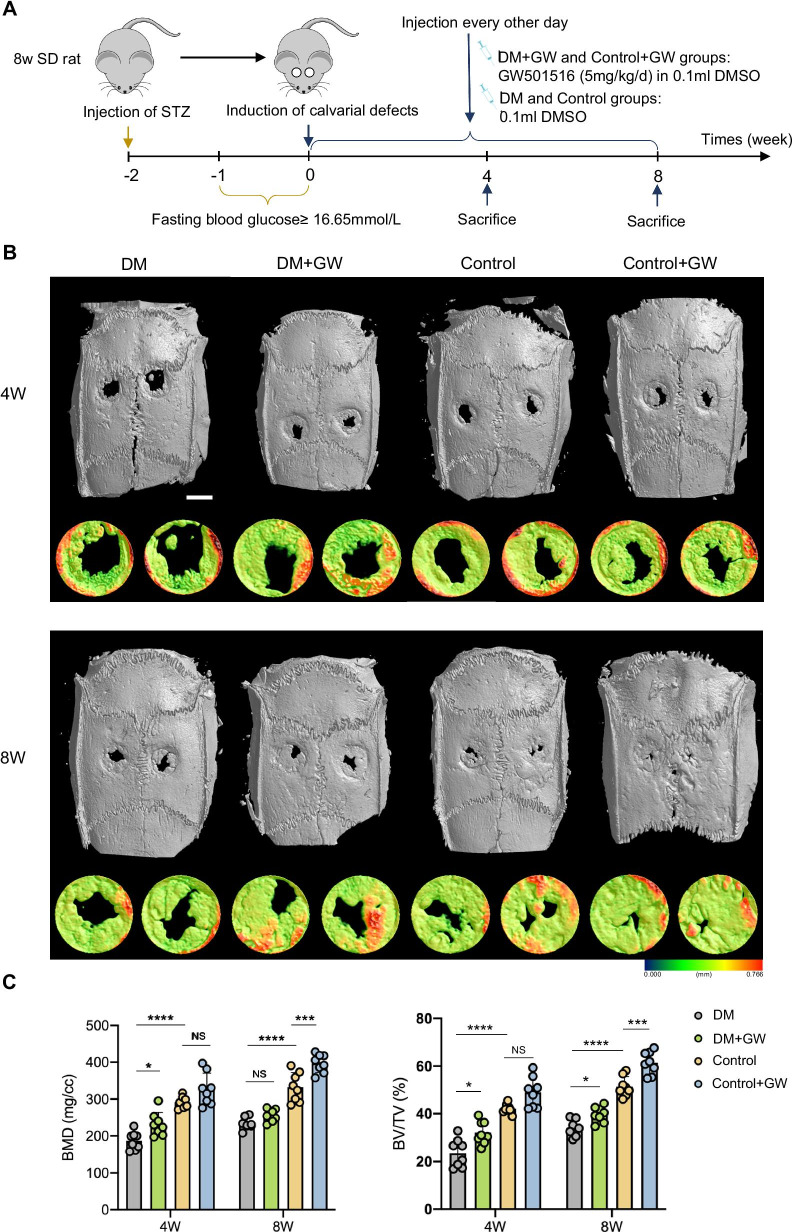
PPARβ/δ agonist accelerates bone regeneration of calvarial defects in diabetic rats. **A** Schematic illustration of experimental design. **B** Representative images of μCT regeneration of carvarial defects at 4 and 8 weeks (scale bar = 3 mm). **C** Quantitative μCT analyses of bone mineral density (BMD, mg/cc) and bone volume/total volume (BV/TV, %) within the original defect position boundary (n = 8). Data are expressed as mean ± SD. The *p* values were calculated by two-way ANOVA with Tukey’s post hoc test. (NS, not statistically significant, *p < 0.05, ***p < 0.001, ****p < 0.0001)

**Fig. 3:**
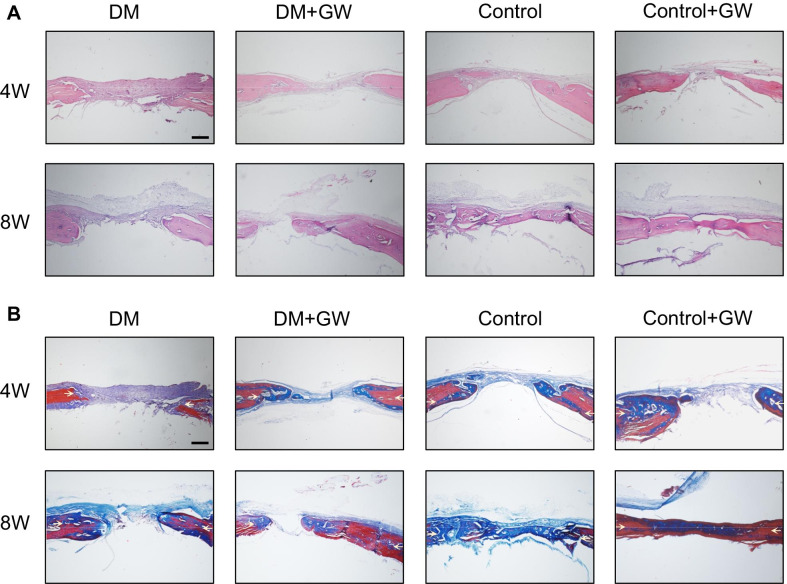
PPARβ/δ agonist promotes the formation of new bone and collagen of calvarial defects in diabetic rats. **A** Representative images of H&E staining of calvarial bone sections. **B** Representative images of Masson staining of calvarial bone sections. In the defect area, blue indicates new bone and collagen (scale bar = 300 μm). The calvarial defect margins are indicated by arrows

We explored the effects of PPARβ/δ agonist on the bone regeneration of rat calvarial defects (Fig. 2A). Through μCT analysis of calvarial defects, we found that both BMD and BV/TV were reduced in diabetic rats compared with the control, which were significantly increased after injection of GW501516 (Fig. 2B, C). H&E and Masson staining showed that diabetes mellitus inhibited the formation of new bone and collagen around the calvarial defects, which were promoted by GW501516 (Fig. 3A, B).

### The activation of PPARβ/δ in high-glucose environment promotes the autophagy and up-regulates osteogenic pathways

**Fig. 4:**
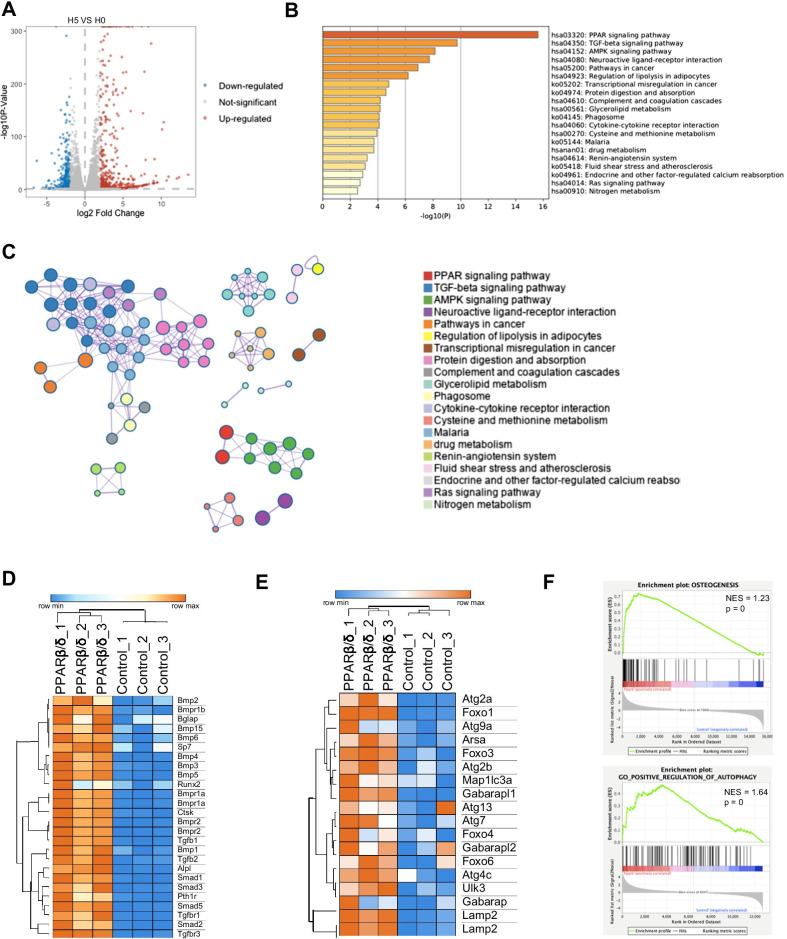
RNA sequencing shows PPARβ/δ agonist promotes the up-regulation of autophagy and osteogenic pathways in high-glucose environment. **A** The volcano plot shows that 684 genes up-regulated and 420 genes down-regulated in H5 group compared with H0 group. **B** KEGG enrichment analysis shows the signaling pathways up-regulated after GW501516 treatment. **C** KEGG network diagram shows the interaction between the up-regulated signal pathways after GW501516 treatment. **D** Heatmap of representative osteogenesis associated genes intensively expressed in H5 group compared with H0 group. **E** Heatmap of representative autophagy associated genes intensively expressed in H5 group compared with H0 group. **F** GSEA showed increased enrichment of osteogenic differentiation and autophagy-related regulatory genes after GW501516 treatment

RNA sequencing analysis was performed on rBMSCs after 7 days of osteogenic induction in high-glucose environment. Compared to H0 group, H5 group had 684 up-regulated genes and 420 down-regulated genes (Fig. 4A). KEGG functional enrichment analysis demonstrated that the PPAR-related pathway and AMPK pathway were significantly up-regulated in H5 group when compared to H0 group, KEGG network diagram showed the close interaction between the PPAR signaling pathway and the AMPK signaling pathway after GW501516 treatment (Fig. 4B, C). The expression of osteogenic marker genes and autophagy pathway-related genes was up-regulated in H5 group (Fig. 4D, E). GSEA enrichment analysis revealed that the osteogenic differentiation and autophagy-related regulatory genes of rBMSCs were up-regulated by PPARβ/δ agonist in high-glucose environment (Fig. 4F).

### PPARβ/δ agonist promotes AMPK/mTOR-mediated autophagy in high-glucose environment

**Fig. 5:**
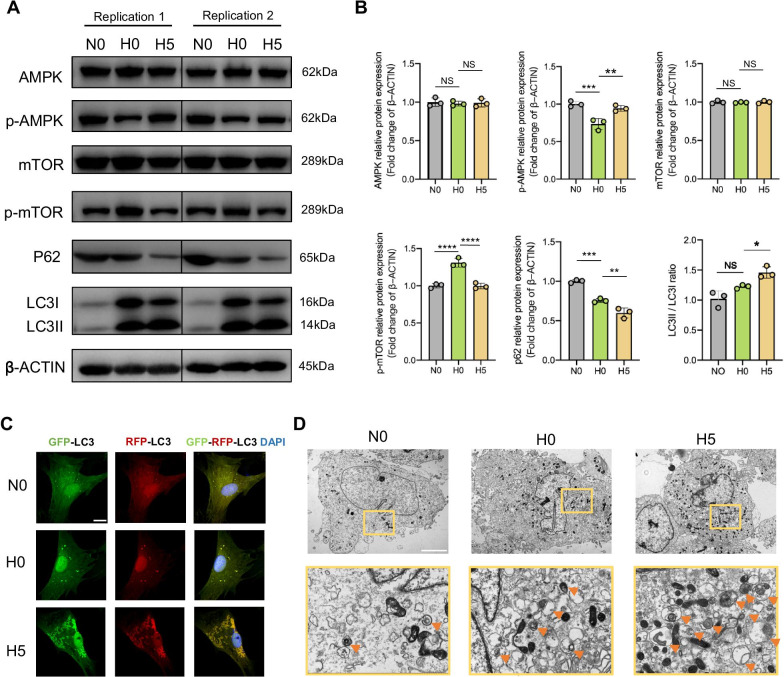
PPARβ/δ agonist promotes AMPK/mTOR-mediated autophagy in high-glucose environment. **A** AMPK, p-AMPK, mTOR, p-mTOR, LC3 and p62 were resolved via western blot after N0, H0 and H5 groups cultured for 7 days of osteogenic induction. β-ACTIN was the loading control. **B** Gray values of bands of AMPK, p-AMPK, mTOR, p-mTOR, LC3 and p62. **C** LSCM images of rBMSCs transfected with RFP-GFP-LC3 adenovirus. DAPI labeled the nuclei (scale bar = 16 μm). The merge number of RFP and GFP dot formation (yellow) indicated the autophagy level. **D** TEM images of rBMSCs after 7 days of osteogenic induction in high-glucose environment (scale bar = 50 μm). Typical autophagosomes are indicated by arrows. Data are expressed as mean ± SD. The *p* values were calculated by one-way ANOVA with Tukey’s post hoc test. (NS, not statistically significant, *p < 0.05, **p < 0.01, ***p < 0.001, ****p < 0.0001)

The expression of p-AMPK was decreased while p-mTOR was increased in H0 group. The addition of GW501516 enhanced p-AMPK level and reduced p-mTOR level. Compared to N0 group, the reduction in p62 expression and increase in LC3-II/LC3-I ratio were detected in H0 group, both of which were amplified in H5 group (Fig. 5A, B), the increase in the ratio of LC3-II/LC3-I and the decrease in p62 protein level can reflect the activation in autophagy [56]. The observation from LSCM and TEM showed that the number of autophagosomes was enhanced in H0 group, which was further elevated in H5 group (Fig. 5C, D).

### PPARβ/δ agonist promotes osteogenic differentiation of rBMSCs through AMPK/mTOR-regulated autophagy

**Fig. 6:**
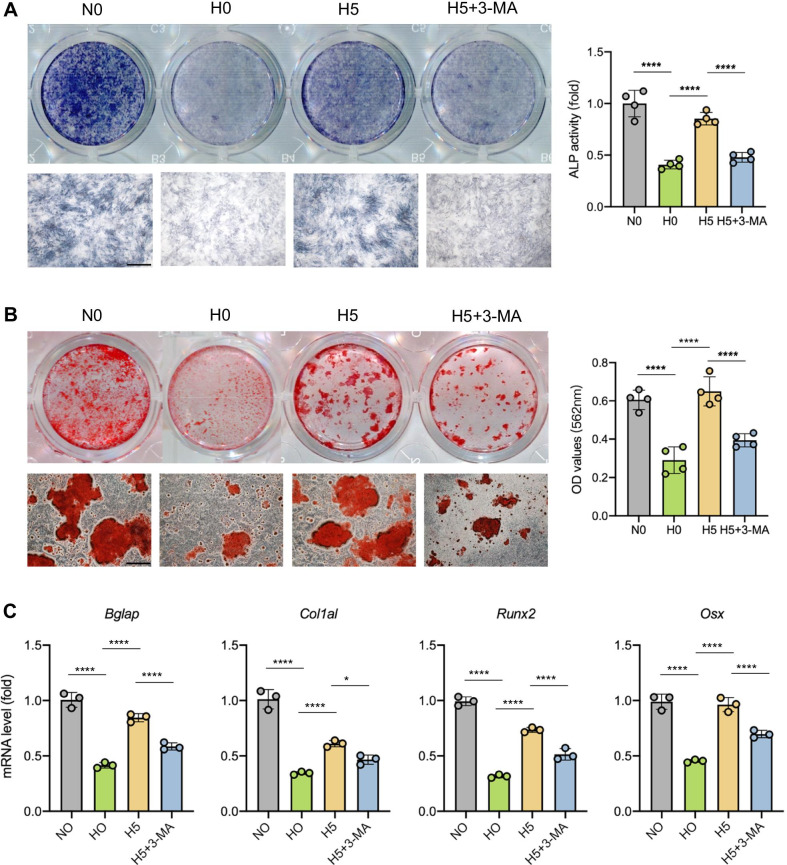
Autophagy promotes osteogenic differentiation of rBMSCs in high-glucose environment. **A** ALP staining and ALP activity quantitative analyses of N0, H0, H5 and H5 + 3-MA groups cultured for 7 days of osteogenic induction. **B** ARS staining and quantitative analyses of rBMSCs after 21 days of osteogenic induction. **C** qRT-PCR for the osteogenesis-related genes *Runx2*, *Bglap*, *Col1a1* and *Osx* after 7 days of osteogenic induction. Data are expressed as mean ± SD. The p values were calculated by one-way ANOVA with Tukey’s post hoc test (*p < 0.05, ****p < 0.0001)

We used 3-Methyladenine (3-MA), a specific autophagy inhibitor, to identify the involvement of autophagy in GW501516-mediated osteogenic differentiation. The degree of ALP staining and mineralized nodules of ARS staining was decreased by adding 3-MA. The increased osteogenic differentiation by GW501516 was also partially inhibited (Fig. 6A, B). Compared to H5 group, the mRNA expressions of osteogenic markers were decreased by adding 3-MA (Fig. 6C). Then, we used AMPK inhibitor Compound C to further investigate the connection between PPARβ/δ and AMPK signal pathway. The ALP staining degree of rBMSCs in GW501516 + Compound C (H5 + Compound C) group was significantly decreased. And the addition of Compound C inhibited mRNA expressions of osteogenesis-related genes (Additional file 1: Figure S4A-C).

## Discussion

The long-term exposure to diabetes mellitus disturbs bone metabolism and leads to impaired bone quality, which predispose diabetic patients to higher fracture risk and slower osseus healing [6]. Currently, it is still clinically challenging to achieve ideal bone healing in diabetic patients [9]. Recent studies revealed that PPARβ/δ served as a critical regulator of bone metabolism and promoted the osteogenic differentiation of MSCs. Additionally, the use of PPARβ/δ agonists has been shown to alleviate the progression of diabetic mellitus [37–39]. Therefore, the present study investigated the effects of PPARβ/δ agonists on the osteogenic differentiation of rBMSCs in vitro and on the bone healing of calvarial defects in vivo under high-glucose condition.

Consistent with previous studies [40], the osteogenic differentiation of rBMSCs was inhibited in high-glucose environment in our study, while the inhibitory effect was partially reversed by PPARβ/δ agonist GW501516 in a concentration-dependent manner. In addition, the delayed bone regeneration of calvarial defects in diabetic rats was also promoted when treated with GW501516. The beneficial effects of PPARβ/δ on bone formation were recently revealed. In normal-glucose environment, PPARβ/δ enhanced the osteogenic differentiation of MSCs via Wnt signal pathway and restored bone density in a mouse model of postmenopausal osteoporosis [19]. The PPARβ/δ-induced osteogenesis was also related to peroxisome expression and redox balance [22]. However, the mechanisms of PPARβ/δ-mediated osteogenesis in high-glucose environment remained unclear. Hence, we performed RNA sequencing to explore the mechanisms of PPARβ/δ-mediated osteogenesis in hyperglycemia condition. The KEGG analysis found that the AMPK signaling pathway was up-regulated by GW501516, and the subsequent GESA analysis suggested that both osteogenesis and autophagy activity were up-regulated. The involvement of AMPK pathway and autophagy in osteogenesis has been well documented [41–43]. Briefly, AMPK is a critical signal molecule that maintains the homeostasis of cell metabolism [44]. The activation of AMPK inhibits mTOR phosphorylation, thereby triggering autophagy [45–49]. Autophagy regulates the stemness of MSCs and is a necessary process in osteogenic differentiation and mineralization [50]. Hence, the PPARβ/δ-induced osteogenesis in high-glucose environment is likely to be caused by the AMPK pathway-mediated autophagy.

Subsequently, we investigated the expression of autophagy-related protein and formation of autophagosomes. It was not surprising to detect enhanced autophagy in HG group. As an adaptive response, autophagy can be activated by various pathological environments to maintain cellular homeostasis and clear damaged organelles and misfolded proteins via autolysosome degradation pathway [51]. However, the reduction in p-AMPK level and the resulting increase in p-mTOR level in HG group did not lead to the inhibition of autophagy, indicating that the high-glucose condition suppressed the activation of AMPK, and the AMPK/mTOR axis was not the critical regulator of autophagy in high-glucose environment without other interventions. Indeed, numerous studies found that high-glucose condition enhanced autophagy while reducing p-AMPK and increasing p-mTOR [42, 52–55]. In this study, the addition of GW501516 greatly enhanced p-AMPK level, yielding decreased mTOR level, which further increased autophagy. This finding was consistent with previous studies demonstrating the regulatory effect of AMPK/mTOR pathway on autophagy [56, 57]. These results also demonstrated that the activation of PPARβ/δ promoted autophagy in MSCs in high-glucose environment via AMPK/mTOR pathway.

3-Methyladenine (3-MA) was used to further verify whether the autophagy was involved in the PPARβ/δ-induced osteogenesis in high-glucose environment. 3-MA is an effective inhibitor of autophagy by blocking autophagy vesicle initiation-related PI3K/AKT pathway [58]. The osteogenic effect of cells under GW501516 treatment in high-glucose condition was suppressed by 3-MA, which proved the involvement of autophagy in PPARβ/δ-induced osteogenesis. By administering the AMPK inhibitor Compound C, we investigated the role of AMPK signaling pathway in PPARβ/δ-mediated osteogenic differentiation. Inhibition of AMPK almost completely eliminated the restored osteogenic differentiation induced by PPARβ/δ agonist under high-glucose condition. Taken together, it seems reasonable to speculate that PPARβ/δ promoted the osteogenesis in high-glucose environment via AMPK/mTOR-mediated autophagy.

## Conclusion

The present study demonstrates that the activation of PPARβ/δ can improve the osteogenic differentiation of rBMSCs in high-glucose environment and promote the healing of calvarial defects in diabetic rats for the first time. To summarize, the PPARβ/δ activation up-regulates AMPK phosphorylation, which leads to mTOR suppression and enhanced autophagy activity. Thus, the impaired osteogenic differentiation of MSCs in high-glucose condition can be restored. Our data suggest PPARβ/δ to be a potential molecular target for the treatment of impaired bone quality and delayed bone regeneration in diabetic patients.

## Supplementary Information

**Additional file 1:** **Figure S1**. The rBMSCs characterization was performed by three-line differentiation and flow cytometry. (A) ARS staining after osteogenic induction (scale bar = 500 µm). (B) Alcian Blue staining after chondrogenic induction (scale bar = 50 µm) (C) Oil Red O after adipogenic induction (scale bar = 50 µm) (D) rBMSCs were CD29/CD44 positive and CD34/CD45 negative. **Figure S2**. The effects of PPARβ/δ agonist (GW0742) and antagonist (GSK0660) on the osteogenic differentiation of rBMSCs in normal- and high-glucose conditions. (A, B) ALP staining and ALP activity quantitative analyses of rBMSCs after 7 days of osteogenic induction treated with 5µM GW0742 or GW501516 (scale bar = 500 µm). (C) qRT-PCR for the osteogenesis-related genes *Runx2*, *Bglap*, *Col1a1* and *Osx* after 7 days of osteogenic induction. (D) qRT-PCR for *Bmp2* after 7 days of osteogenic induction. (E, F) ALP staining and ALP activity quantitative analyses of rBMSCs after 7 days of osteogenic induction treated with 1µM GSK0660 (scale bar = 500 µm). (G) qRT-PCR for the osteogenesis-related genes *Runx2*, *Bglap*, *Col1a1* and *Osx* after 7 days of osteogenic induction. Data are expressed as mean ± SD. The *p* values were calculated by two-way ANOVA with Tukey’s post hoc test. (NS, not statistically significant, *p < 0.05, **p < 0.01, ***p < 0.001, ****p <0.0001). **Figure S3**. The effect of GW501516 on osteoclast differentiation of bone marrow-derived macrophage (BMDM) in normal- and high-glucose conditions. (A) TRAP staining for osteoclast differentiation before and after PPARβ/δ agonist treatment (Scale bar = 100 μm). (B) Statistics on the number and size of osteoclasts. Data were expressed as mean ± SD. The *p* values were calculated by two-way ANOVA with Tukey’s post hoc test. (NS, not statistically significant). N.Oc = number of osteoclasts. **Figure S4**. The effects of AMPK inhibitor (Compound C) plus PPARβ/δ agonist (GW501516) on the osteogenic differentiation of rBMSCs in high-glucose conditions. (A, B) ALP staining and ALP activity quantitative analyses of rBMSCs after 7 days of osteogenic induction treated with 5µM GW501516 + 1µM Compound C (scale bar = 500 µm). (C) qRT-PCR for the osteogenesis-related genes *Runx2*, *Bglap*, *Col1a1*, and *Osx* after 7 days of osteogenic induction. Data are expressed as mean ± SD. The *p* values were calculated by two-way ANOVA with Tukey’s post hoc test. (**p < 0.01, ***p < 0.001, ****p <0.0001).

**Additional file 2.** The primer sequences for qRT-PCR.

## Data Availability

The datasets used during the current study are available from the corresponding authors on reasonable request.

## Abbreviations

PPARβ/δ: Peroxisome proliferators-activated receptor β/δ
rBMSCs: Rat bone marrow mesenchymal stem cells
ALP: Alkaline phosphatase
ARS: Alizarin red S
qRT-PCR: Quantitative reverse transcription polymerase chain reaction
DM: Diabetes mellitus group
DM + GW: Diabetes mellitus treated with PPARβ/δ agonist GW501516 group
Control: Control group
Control + GW: Control treated with GW501516
NG: Normal glucose
HG: High glucose
N0: 5.6 mM glucose
N1: 5.6 mM glucose + 1 μM GW501516
N5: 5.6 mM glucose + 5 μM GW501516
H0: 30 mM glucose
H1: 30 mM glucose + 1 μM GW501516
H5: 30 mM glucose + 5 μM GW501516
LSCM: Laser confocal microscope
TEM: Transmission electron microscope
μCT: Micro-computed tomography
AMPK: Adenosine 5′-monophosphate-activated protein kinase
mTOR: Mammalian target of rapamycin
MSC: Mesenchymal stem cell
ROS: Reactive oxygen species
PPREs: Peroxisome proliferator response elements
SD: Sprague–Dawley
STZ: Streptozotocin
DMSO: Dimethyl sulfoxide
ROI: Region of interest
BMD: Bone mineral density
BV/TV: Bone volume/total volume
GAPDH: Glyceraldehyde 3-phosphate dehydrogenase
RUNX2: Runt-related transcription factor 2
Bglap: Bone gamma-carboxyglutamate (gla) protein
Col1a1: Collagen, type I, alpha 1
Osx: Osterix
H&E: Hematoxylin and eosin
EDTA: Ethylene diamine tetraacetic acid
DMEM: Dulbecco's modification of Eagle's medium
RIPA: Radio-immunoprecipitation assay
SDS-PAGE: Sulfate–polyacrylamide gel electrophoresis
RNA-seq: RNA sequencing
KEGG: Kyoto encyclopedia of genes and genomes
GSEA: Gene set enrichment analysis
ANOVA: Analysis of variance
3-MA: 3-Methyladenine
SD: Standard deviation
