# Supplementary figures and images for: PPARβ/δ accelerates bone regeneration in diabetic mellitus by enhancing AMPK/mTOR pathway-mediated autophagy
Source: Stem Cell Res Ther. 2021 Nov 4;12:566. doi: 10.1186/s13287-021-02628-8 (PMC8567548; doi:10.1186/s13287-021-02628-8)

**Figure S1**

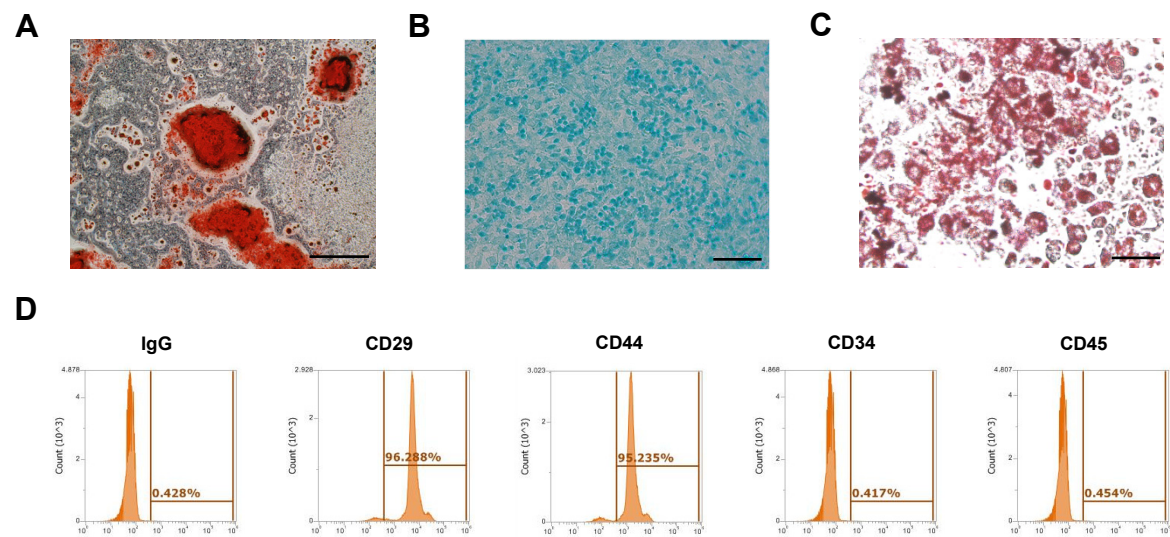

Figure S2

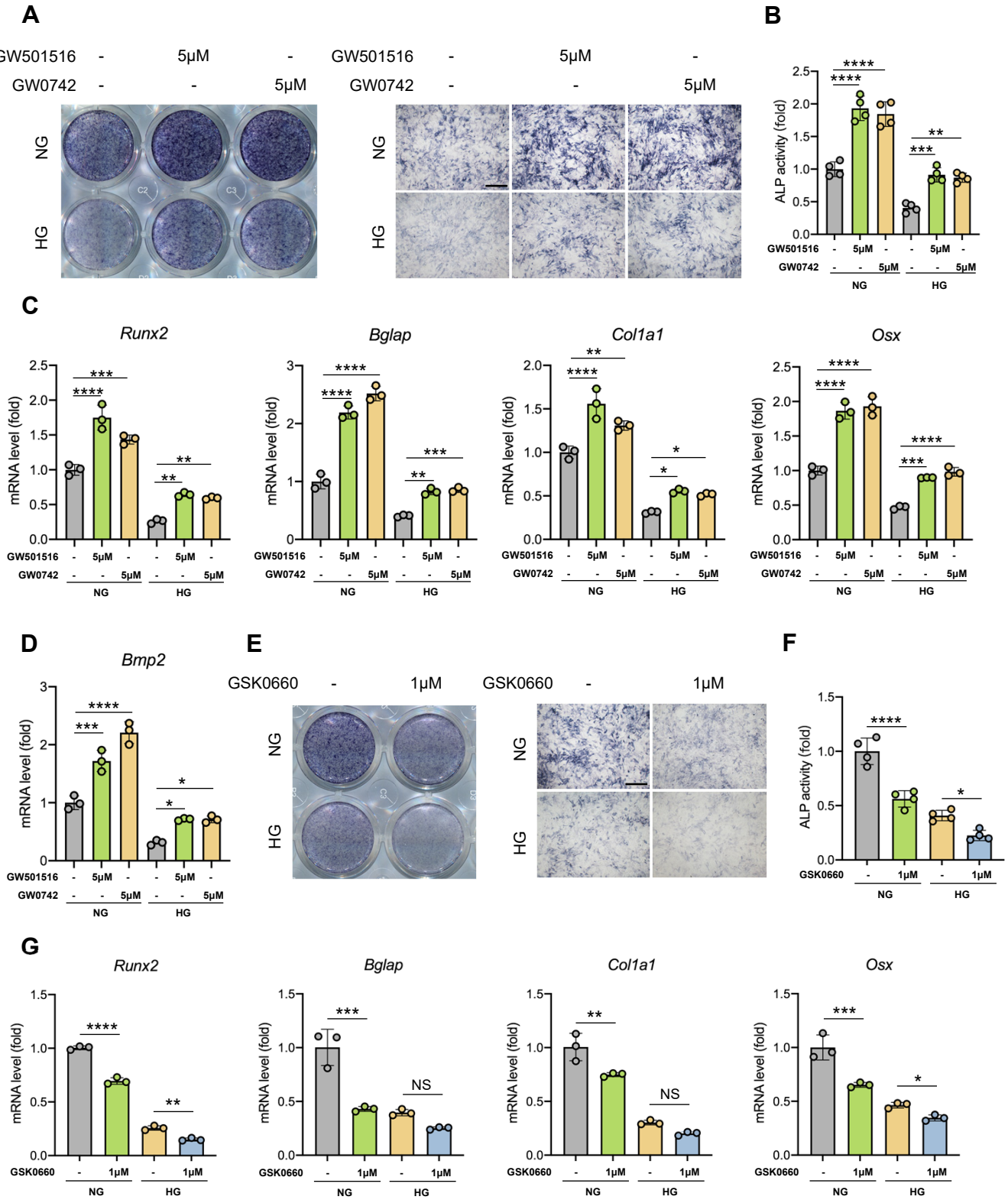

Figure S3

A

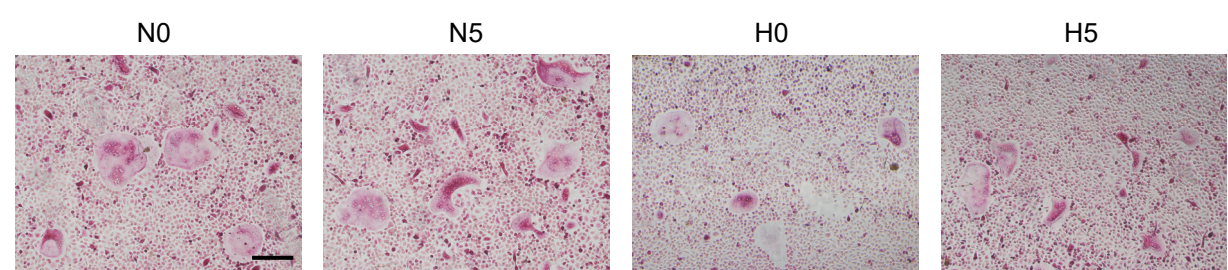

B

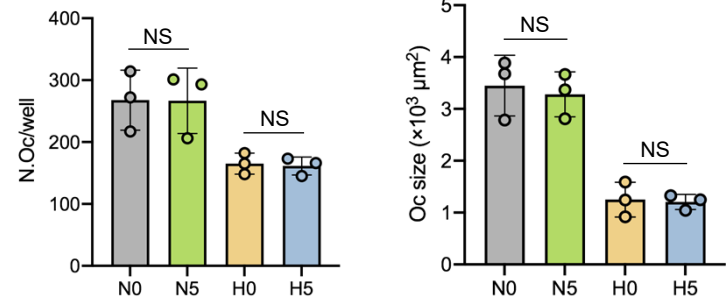

Figure S4

A

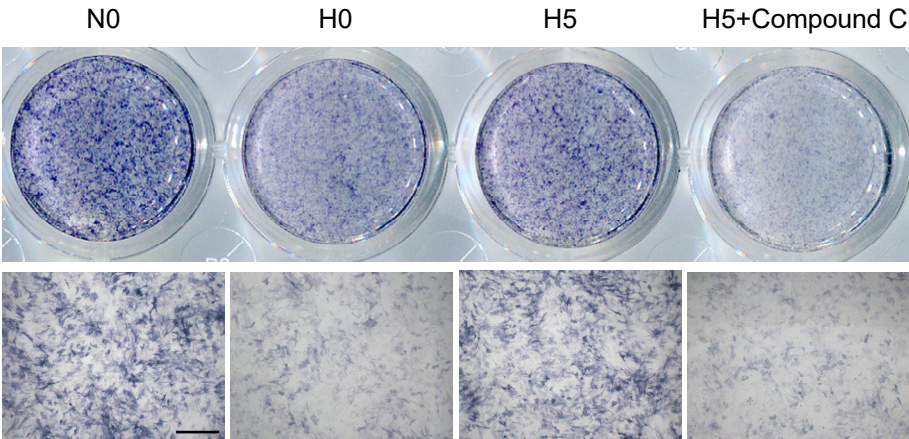

B

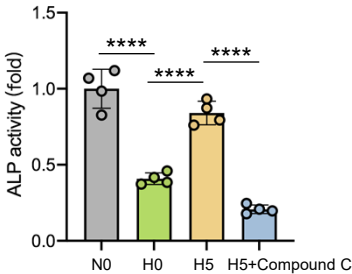

C

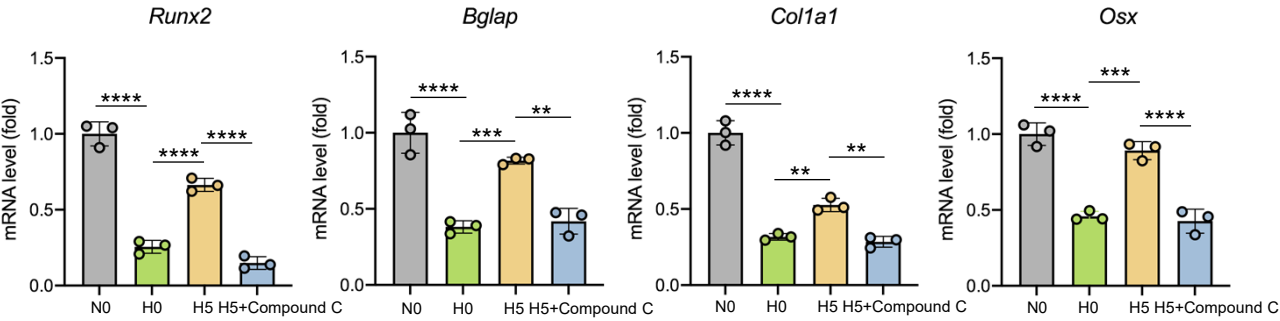

Supplement: Supplementary file 1 — Additional file 1: Figure S1. The rBMSCs characterization was performed by three-line differentiation and flow cytometry. (A) ARS staining after osteogenic induction (scale bar = 500 µm). (B) Alcian Blue staining after chondrogenic induction (scale bar = 50 µm) (C) Oil Red O after adipogenic induction (scale bar = 50 µm) (D) rBMSCs were CD29/CD44 positive and CD34/CD45 negative. Figure S2. The effects of PPARβ/δ agonist (GW0742) and antagonist (GSK0660) on the osteogenic differentiation of rBMSCs in normal- and high-glucose conditions. (A, B) ALP staining and ALP activity quantitative analyses of rBMSCs after 7 days of osteogenic induction treated with 5µM GW0742 or GW501516 (scale bar = 500 µm). (C) qRT-PCR for the osteogenesis-related genes Runx2, Bglap, Col1a1 and Osx after 7 days of osteogenic induction. (D) qRT-PCR for Bmp2 after 7 days of osteogenic induction. (E, F) ALP staining and ALP activity quantitative analyses of rBMSCs after 7 days of osteogenic induction treated with 1µM GSK0660 (scale bar = 500 µm). (G) qRT-PCR for the osteogenesis-related genes Runx2, Bglap, Col1a1 and Osx after 7 days of osteogenic induction. Data are expressed as mean ± SD. The p values were calculated by two-way ANOVA with Tukey’s post hoc test. (NS, not statistically significant, *p < 0.05, **p < 0.01, ***p < 0.001, ****p <0.0001). Figure S3. The effect of GW501516 on osteoclast differentiation of bone marrow-derived macrophage (BMDM) in normal- and high-glucose conditions. (A) TRAP staining for osteoclast differentiation before and after PPARβ/δ agonist treatment (Scale bar = 100 μm). (B) Statistics on the number and size of osteoclasts. Data were expressed as mean ± SD. The p values were calculated by two-way ANOVA with Tukey’s post hoc test. (NS, not statistically significant). N.Oc = number of osteoclasts. Figure S4. The effects of AMPK inhibitor (Compound C) plus PPARβ/δ agonist (GW501516) on the osteogenic differentiation of rBMSCs in high-glucose conditions. (A, B) ALP s [file 13287_2021_2628_MOESM1_ESM.pdf]
