# Supplementary material for: PPARβ/δ accelerates bone regeneration in diabetic mellitus by enhancing AMPK/mTOR pathway-mediated autophagy
Source: Stem Cell Res Ther. 2021 Nov 4;12:566. doi: 10.1186/s13287-021-02628-8 (PMC8567548; doi:10.1186/s13287-021-02628-8)
Supplement: Supplementary file 2 — Additional file 2. The primer sequences for qRT-PCR. [file 13287_2021_2628_MOESM2_ESM.docx]

Additional file 2. The primer sequences for qRT-PCR.

| qRT-PCR (rat) | |  |
| --- | --- | --- |
| Genes | **Primers** | **Sequences (5*’*‐3*’*)** |
| *Runx2* | Forward | CTCTTCCCAAAGCCAGAGCG |
|  | Reverse | ACCATCCTGGAAGGAGACCG |
| *Bglap* | Forward | AGACTCCGGCGCTACCTCAAC |
|  | Reverse | GGCGTCCTGGAAGCCAATGTG |
| *Col1a1* | Forward | TGTTGGTCCTGCTGGCAAGAATG |
|  | Reverse | GTCACCTTGTTCGCCTGTCTCAC |
| *Osx* | Forward | GCCTACTTACCCGTCTGACTTTGC |
|  | Reverse | CCCTCCAGTTGCCCACTATTGC |
| *Bmp2* | Forward | GCAAAGAAAAGGAACGGACATT |
|  | Reverse | GGGAAGCAGCAACGCTAGAA |
| *Gapdh* | Forward | GGGTGTGAACCACGAGAAAT |
|  | Reverse | ACTGTGGTCATGAGCCCTTC |
